# Modelling brain metabolism with interacting nonautonomous phase oscillators

**DOI:** 10.3389/fnetp.2026.1720336

**Published:** 2026-02-20

**Authors:** Samuel J. K. Barnes, Anaí Echeverría, Joshua Hawley, Yevhen F. Suprunenko, Aneta Stefanovska

**Affiliations:** 1 Department of Physics, Lancaster University, Lancaster, United Kingdom; 2 VIB-Neuro-Electronics Research Flanders, Leuven, Belgium; 3 Department of Biology, KU Leuven, Leuven, Belgium; 4 Faculty of Electrical Engineering, University of Ljubljana, Ljubljana, Slovenia

**Keywords:** astrocyte, brain, coupled oscillators, metabolism, network physiology, neurovascular unit, nonautonomous systems, phase dynamics

## Abstract

Traditional brain models have focused primarily on electrical signalling, offering valuable insights but often overlooking the crucial role of metabolism within the neurovascular unit. Existing metabolic models tend to be highly detailed and mass-based, relying on strict conservation laws that limit their applicability to the brain’s thermodynamically open environment. In this study, we present a novel, phenomenological model of neuronal energy metabolism using a network of coupled Kuramoto oscillators. This nonautonomous phase dynamics framework captures complex, time-dependent interactions and allows for multiple synchronization states among metabolic processes. Our model captures key features consistent with healthy neurovascular dynamics, despite not being directly fitted to empirical data from resting-state brains and reveals how disruptions in metabolic synchrony may contribute to dementia-related pathology. By emphasizing the importance of metabolic coordination in the neurovascular unit, this work provides a versatile methodological foundation for future brain modelling efforts.

## 1 Introduction

The human brain is often described as one of the most complex systems in the universe (Haken, 1983a). Most efforts to characterise brain function have focused on neuronal activity (Jirsa and Haken, 1996; Haken, 2015). Mathematical models, ranging from integrate-and-fire (Burkitt, 2006; Haken, 2008; Kobayashi et al., 2019; Chauhan et al., 2024), to models of higher order connectomes (Santoro et al., 2024) and digital twins of the human brain function (Jirsa et al., 2023), have provided effective descriptions of electrical activity. In parallel, alternative models have established important links between regional metabolites, such as GABA and glutamate at the mesoscopic scale, and large-scale blood oxygen level-dependent (BOLD) activity measured with fMRI (Naskar et al., 2021; Luppi et al., 2022). These frameworks have substantially advanced our understanding of how local neuronal and neurochemical processes relate to large-scale brain dynamics. However, they typically treat metabolic variables in a kinetic or time-independent manner. As a result, the time-dependent dynamic supply and utilisation of metabolic substrates, such as glucose and oxygen, required to sustain ongoing brain activity remain incompletely represented.

Despite major advances in understanding brain function, several fundamental questions remain unresolved, including how neuronal activity is dynamically constrained by metabolic availability, how energy supply adapts to sustained or pathological neural demand, and how failures of these regulatory mechanisms give rise to disease. Although the brain constitutes only approximately 2% of body mass, it accounts for nearly 20% of resting energy expenditure, underscoring the critical importance of tightly regulated substrate delivery (Iadecola, 2017). This regulation is mediated by the neurovascular unit (NVU), a functional ensemble of neurons, astrocytes, endothelial cells, and vascular smooth muscle that together maintain cerebral metabolism and perfusion (Alkayed and Cipolla, 2023). Central to the NVU is neurovascular coupling (NVC), a bidirectional process in which neural activity modulates cerebral blood flow, while metabolic supply in turn constrains neuronal dynamics. Disruptions of NVC are increasingly recognised in ageing, dementia, and other neurological conditions (Bjerkan et al., 2023; Bjerkan et al., 2024; Bjerkan et al., 2025; Shichkova et al., 2025).

The growing recognition of the NVU has motivated detailed models of cellular and regional brain energy metabolism (Shichkova et al., 2025; Somersalo et al., 2012; Sundqvist et al., 2022). While these models provide important mechanistic insight, they are often highly complex and rely on large numbers of parameters. Moreover, they are typically mass-based, enforcing strict substrate conservation, even though living systems are thermodynamically open (Von Bertalanffy, 1950). Consequently, the dynamical principles governing collective metabolic regulation across interacting cellular components remain poorly understood.

We present a model of neuronal energy metabolism grounded in the principles of synergetics (Haken, 1983b) and the theory of interacting Kuramoto oscillators (Kuramoto, 1984). The model operates at a cellular scale by highlighting the interactions between metabolic units. Each unit is described as a nonlinear, time-dependent oscillator, with bidirectional interactions capturing the complex interplay between neuronal demand, astrocytic support, and vascular supply. This framework employs relatively few parameters while explicitly representing the system as nonlinear, time-dependent, and thermodynamically open (Lancaster et al., 2016; Rowland Adams and Stefanovska, 2021). The approach builds on the nonautonomous phase dynamics framework (Kloeden and Rasmussen, 2011; Suprunenko et al., 2013; Rowland Adams et al., 2023), which is intrinsically suited for the treatment of such systems. Within this framework, synchronisation refers to phase locking and coordinated temporal organisation between interacting metabolic and neurovascular oscillations, rather than neuronal spike synchrony. We demonstrate how alterations in metabolic synchronisation can destabilise energy supply, with the model reproducing dementia-like dynamics that can be contrasted with those observed in a healthy brain.

The paper is structured as follows. Section 2 formulates the model by identifying relevant metabolic oscillators and their interactions. Section 3 specifies parameter space defining healthy and dementia states. Section 4 presents dynamic transitions in the healthy model under increasing neural load, followed by cases of impaired substrate supply, including reduced astrocytic lactate output, reduced glucose availability, and reduced oxygen delivery. Sections 5, 6 discuss the implications and summarise the findings.

## 2 The model

The neurovascular unit comprises multiple interacting components that together form a complex network of coupled oscillators. Understanding the dynamics of the unit as a whole requires consideration of how each element influences, and is influenced by, the others. In this section, these interacting components are examined, and their mutual effects are quantified to construct a model that captures the emergent phenomena arising from their couplings.

### 2.1 The biological background

The brain is one of the most metabolically active organs in the body, and its energy usage is tightly coupled to neural performance (Simon, 2001). Therefore, employing appropriate pathways to synthesize ATP, the body’s primary energy currency, is paramount. Alterations to the pathways responsible for ATP synthesis lead to various states of disease (Liu et al., 2025).

In mammals, cellular energy metabolism can be summarized into four key processes. The first stage, glycolysis, occurs in the cytoplasm, where glucose is converted into pyruvate. This process produces two molecules of adenosine triphosphate (ATP) and reduces nicotinamide adenine dinucleotide ( ${\text{NAD}}^{+}$ ) to NADH. Next, in the mitochondria, oxidative phosphorylation (OXPHOS) completes metabolism so efficiently that 28 molecules of ATP are synthesised per glucose molecule. OXPHOS utilises NADH and pyruvate from glycolysis, and oxygen, which diffuses across cell membranes. Glucose is delivered to the cytoplasm for glycolysis via glucose transporter proteins (GLUTs), which facilitate membrane diffusion (Koepsell, 2020).

**FIGURE 1:**
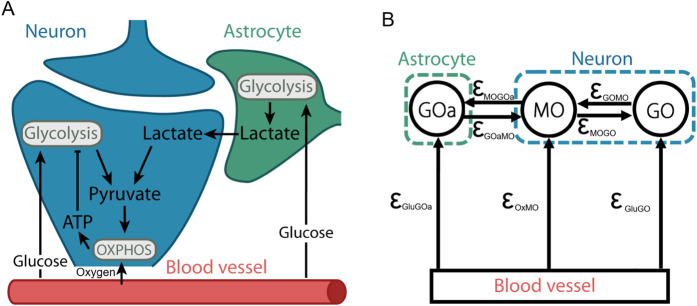
Biochemical interactions and metabolic processes within the neurovascular unit **(A)** can be abstracted as a system of coupled metabolic oscillators **(B)**. The solid black nodes in **(B)** represent specific oscillatory processes mapped from **(A)** ${\text{GO}}_{a}$ corresponds to the astrocyte glycolytic oscillator, GO to neuronal glycolytic oscillator, MO to neuronal mitochondrial oscillator - where oxidative phosphorylation (OXPHOS) takes place. The blood vessel represents glucose and oxygen supply. ϵ represents coupling strengths between oscillators.

Recently, the essential role of glial cells in the brain has been highlighted (Afridi et al., 2020). Astrocytes support cellular energy metabolism by producing lactate via glycolysis (Gandhi et al., 2009). This lactate is then shuttled into the neurons via monocarboxylate transporters (MCTs) (Yamagata, 2022) and used in the Krebs cycle within the mitochondria (Barros and Weber, 2018; Oliver, 2024). The interplay between these processes is illustrated in Figure 1.

### 2.2 Response to high energy demand

The brain is an inherently nonautonomous dynamical system, as its energy demand continuously changes depending on the cognitive demand at any given moment. Metabolic flexibility is therefore essential to maintain health (Chausse et al., 2024). At rest, the brain consumes glucose and oxygen optimally to metabolise ATP using oxidative phosphorylation (Fox et al., 1988; Lund Madsen et al., 1999). Glucose uptake in the brain increases substantially more than the increase in oxygen during periods of stimulation, as demonstrated using positron emission tomography (Fox et al., 1988). This adaptability is essential to maintain proper brain function and respond to a changing environment (Chausse et al., 2024). Additionally, recent evidence suggests neuronal, rather than astrocytic glycolysis, is primarily upregulated during periods of cognitive demand (Manlio Díaz-García et al., 2017). The balance between these mechanisms is debated (Oliver, 2024; Barros and Weber, 2018), hence both are included in the current model.

### 2.3 Metabolic oscillations

Metabolic processes are inherently oscillatory. Mitochondrial function is dictated by oscillations between an oxidative and reductive environment (Aon et al., 2008; Kurz et al., 2010a). These oscillations can be measured by the florescence of NADH (Mayevsky and Rogatsky, 2007) or the mitochondrial membrane potential (Gerencser et al., 2012). Furthermore, glycolysis exhibits oscillatory characteristics regulated by several protein complexes (Olsen et al., 2009). Concentrations of glycolytic intermediates oscillate with a period of a few minutes (Xiong and Garfinkel, 2023) and produce NADH which is measured using florescence (Duysens and Amesz, 1957). When operating optimally, glycolysis provides OXPHOS with sufficient molecular substrates to enable optimal energy metabolism. Additionally, the supply of oxygen to the brain is characterised by a baseline level around which oscillations occur (Daniil et al., 2018). These dynamics are influenced by changes in the vascular tone of arterioles and vary with age (Doubovikov and Aksenov, 2020). The oscillatory nature of cellular energy metabolism necessitates an approach that explicitly treats each component as such. Glycolysis can also take place in astrocytes. This process generates lactate, which is shuttled into the neuron to provide an alternative fuel source for the mitochondria (Theparambil et al., 2024). Lactate transporter proteins called monocarboxylate transporters (MCTs) shuttle this metabolite from the astrocyte to the neuron, supporting oxidative metabolism.

### 2.4 Nonautonomous phase dynamics model

Rather than treating the system as a closed or isolated, here we apply a system of differential equations to simulate the interactions between the metabolic units and external influences. By focussing upon the phase dynamics of these oscillators, we can substantially simplify the model compared to traditional approaches based upon metabolite concentrations, and so require conservation of mass. Instead we present an alternative approach focusing on the interactions summarised in Figure 1. Additionally, Focusing on phase rather than amplitude dynamics substantially enhances the system’s robustness to noise (Barnes et al., 2024).

To incorporate external influences and metabolic couplings, we adopt the Kuramoto model (Kuramoto, 1984; Kuramoto, 1975). Astrocytes, neurons, and blood vessels exhibit oscillatory activity, and the Kuramoto framework captures their synchronization by reducing complex biochemical interactions to essential rhythmic dynamics, enabling the study of timing and coordination across metabolic components. In biological systems, oscillatory frequencies often fluctuate around a central value (Johnston et al., 2020). These fluctuations are partially attributed to environmental perturbations and deterministic influences. To capture this behaviour, we explicitly model the system as nonautonomous, where phase oscillators have time-dependent frequencies. This approach reflects the dynamic characteristics observed in living systems. Specifically, the oscillator frequencies are modulated over time such that:

$$\omega \left(t\right)=\omega \left(1+A\sin\left(\omega_{\text{mod}}t\right)\right),$$ (1)

where $\omega_{\text{mod}}$ represents the modulation frequency, and A is the amplitude of modulation around the central value ω . Somewhat counterintuitively, introducing a deterministic, nonautonomous frequency has been shown to expand regions of stability and enhance robustness in the face of environmental changes (Lucas et al., 2018; Lucas et al., 2019). This resilience is vital in cells to maintain a steady supply of ATP to power neuronal function. Metabolic oscillators are also dependent upon their interactions such as the supply of substrates to the cell. We model the strength of these influences using phase couplings ϵ . For example, in the case of the glycolytic oscillator there are two main influences, the supply of glucose from the blood $\left(+\epsilon_{\mathrm{GluGO}}\right)$ , which is excitatory, and an inhibitory influence from the mitochondria $\left(-\epsilon_{\mathrm{MOGO}}\right)$ , which suppresses glycolysis to the low level nesecary to supply substrates to the mitochondrial oscillator. Pink noise, η(t) , modulated with strength σ is also implemented (due to its 1/f distribution) to replicate the remaining, smaller external influences. As demonstrated in Equation 2, the phase of the glycolytic oscillator is given as,

$${\dot{\varphi }}_{GO}=\omega_{GO}-\varepsilon_{\mathrm{MOGO}}\sin\left(\varphi_{MO}-\varphi_{GO}\right)+\varepsilon_{\mathrm{GluGO}}\sin\left(\varphi_{\mathrm{Glu}}-\varphi_{GO}\right)+\sigma \eta \left(t\right).$$ (2)

Applying this approach to each oscillator demonstrates their evolution and mutual behaviour over time, which will allow us to evaluate which of the components are functionally connected in different circumstances, and ultimately the overall health of the system. Sinusoidal phase couplings provide a robust, efficient, and flexible framework for modelling interactions between rhythmic biological processes (Lancaster et al., 2016). Equation 3 describes the system using its phase dynamics as,

$$\begin{array}{cc} & {\dot{\varphi }}_{Ox}=\omega_{Ox}+\sigma \eta \left(t\right)\\ & {\dot{\varphi }}_{\mathrm{Glu}}=\omega_{\mathrm{Glu}}+\sigma \eta \left(t\right)\\ & {\dot{\varphi }}_{\mathrm{GOa}}=\omega_{\mathrm{GOa}}-\varepsilon_{\mathrm{MOGOa}}\sin\left(\varphi_{MO}-\varphi_{\mathrm{GOa}}\right)+\varepsilon_{\mathrm{GluGOa}}\sin\left(\varphi_{\mathrm{Glu}}-\varphi_{\mathrm{GOa}}\right)\\ & +\sigma \eta \left(t\right)\\ & {\dot{\varphi }}_{GO}=\omega_{GO}-\varepsilon_{\mathrm{MOGO}}\sin\left(\varphi_{MO}-\varphi_{GO}\right)+\varepsilon_{\mathrm{GluGO}}\sin\left(\varphi_{\mathrm{Glu}}-\varphi_{GO}\right)\\ & +\sigma \eta \left(t\right)\\ & {\dot{\varphi }}_{MO}=\omega_{MO}+\varepsilon_{\mathrm{GOMO}}\sin\left(\varphi_{GO}-\varphi_{MO}\right)+\varepsilon_{\mathrm{GOaMO}}\sin\left(\varphi_{\mathrm{GOa}}-\varphi_{MO}\right)\\ & \;+\;\varepsilon_{\mathrm{OxMO}}\sin\left(\varphi_{Ox}-\varphi_{MO}\right)+\sigma \eta \left(t\right). \end{array}$$ (3)

Here, $\omega_{MO}$ is the natural frequency of the mitochondrial oscillator (MO), $\varepsilon_{\mathrm{GOMO}}$ is the coupling from the glycolytic to mitochondrial (GO to MO) due to the effects of neuronal pyruvate on MO, $\varepsilon_{\mathrm{GOaMO}}$ is the coupling from the astrocyte glycolytic oscilator (GOa) to MO due to the effects of astrocytic lactate on MO, $\varphi_{\mathrm{GOa}}$ is the phase of GOa, $\omega_{\mathrm{GOa}}$ is the natural frequency of GOa, $\varepsilon_{\mathrm{GluGO}}$ is the coupling from the substrate to GOa due to the effects of glucose on glycolysis, and $\omega_{Glu}$ and $\omega_{Ox}$ are the natural frequencies of glucose and oxygen, respectively. Positive couplings represent excitatory couplings between oscillators, for example, the supply of glucose to the glycolytic oscillator (GluGO) , while as mentioned previously, mitochondrial activity inhibits glycolytic activity (MOGO) and hence this is represented by a negative, inhibitory coupling.

Because of the nonlinearity of these equations, exact solutions are not available; instead, dynamical analysis or numerical simulations are required to extract useful information.

A fourth-order Runge–Kutta integration scheme was applied to compute the phase values of each oscillator over time, which is well suited for first-order, nonautonomous phase equations of the form considered here. The system was simulated for 2000 s using a time step of 0.1 s. This time step and duration were chosen as it was sufficient to provide stable, convergent results across a series of repeated simulations while not being so small that we approached limits of computational capacity. This scheme provides accurate resolution of phase dynamics and phase relationships in coupled oscillator systems, and has been widely used in previous studies of synchronisation and nonautonomous phase dynamics (Rowland Adams and Stefanovska, 2021; Barnes and Stefanovska, 2021). Alternative integration schemes may also be employed; however, for comparable step sizes, lower-order methods have been shown to be less effective in resolving phase locking and transition dynamics in similar models. The resulting mean phase values of each oscillator were subsequently analysed to determine the presence of synchronization between oscillators. Oscillators were considered synchronised if the range of their phase difference during the latter half of each signal remained below 2π .

## 3 Establishing biologically relevant parameters

Parameter values are selected based upon literature describing the healthy state. The model can then be explored across different parameter sets to simulate metabolic abnormalities. By examining the synchronisation patterns between oscillators and comparing them to expected healthy and altered states, we can construct a model capable of reproducing the qualitative transitions observed in the system. A summary of the parameter values established is provided in Table 1.

**TABLE 1:** Table of parameters for the healthy brain at rest. The arrows indicate the direction of coupling.

Biological correlate	Parameter	Value	References
Oxygen supply freq	$\omega_{ox}$	2π/100 Hz	Daniil et al. (2018)
Glucose supply freq	$\omega_{\mathrm{Glu}}$	2π/200 Hz	Lancaster et al. (2016)
Glycolysis natural freq (neuron)	$\omega_{GO}$	2π/200 Hz	Paul (1995), Merrins et al. (2016), Xiong and Garfinkel (2023)
Glycolysis natural freq (astrocyte)	$\omega_{\mathrm{GOa}}$	2π/200 Hz	Paul (1995), Merrins et al. (2016), Xiong and Garfinkel (2023)
OxPhos natural freq neuron	$\omega_{MO}$	2π/100 Hz	Kurz et al. (2010b), Kurz et al. (2010a)
Oxygen → neuron	$\epsilon_{Ox\to MO}$	0.1	Doubovikov and Aksenov (2020)
Glucose → neuron	$\epsilon_{\mathrm{Glu\to GO}}$	0.1	Mergenthaler et al. (2013)
Glucose → astrocyte	$\epsilon_{\mathrm{Glu\to GOa}}$	0.05	Deitmer et al. (2019)
Lactate → neuron	$\epsilon_{\mathrm{GOa\to MO}}$	0.025	Díaz-García and Yellen (2019), Bonvento and Bolaños (2021)
Glycolysis neuron → oxphos	$\epsilon_{GO\to MO}$	0.1	Yellen (2018)
Glycolysis neuron ← oxphos	$\epsilon_{MO\to GO}$	0.2	Yellen (2018)

### 3.1 Natural frequencies $\omega_{MO}$ , $\omega_{GO}$ and $\omega_{\mathrm{GOa}}$

Natural frequencies of brain metabolic processes are difficult to measure and so parameter values were estimated from known oscillatory frequencies in other cell types. Glycolysis usually takes place on the scale of a few minutes (Xiong and Garfinkel, 2023; Paul, 1995; Merrins et al., 2016) while oxidative phosphorylation is slightly quicker, with a period around 100 s (Kurz et al., 2010a; Kurz et al., 2010a; Vergun and Reynolds, 2004). Given the lack of information about the frequency of glycolysis in the astrocytes and neurons, they were set to the same value $\omega_{GO}=\omega_{\mathrm{GOa}}=\frac{2\pi }{200}$ Hz. These values also correspond to previously implemented nonautonomous phase oscillator metabolic models (Rowland Adams and Stefanovska, 2021; Lancaster et al., 2016; Barnes and Stefanovska, 2021). As outlined in Equation 1 the natural frequencies ω , define a certain basal- or mid-frequency about which oscillations take place.

### 3.2 The parameter space

**FIGURE 2:**
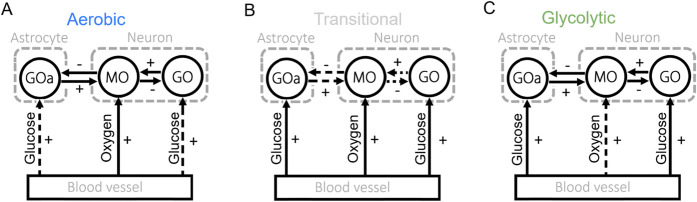
The different metabolic modes explored in the model. **(A)** The aerobic state - driven by the supply of oxygen and relying primarily on oxidative phosphorylation for ATP production. **(B)** Transitional state - no synchronisation between the metabolic oscillators leading to sub-optimal energy metabolism. **(C)** Glycolytic state driven by glucose and/or astrocytic lactate supply.

The relevant aspects of neuronal energy production are outlined in Section 2.1. However, to summarise the expectations for a healthy neuron, we emphasise two key points. First, during resting conditions, oxidative metabolism is the primary pathway for ATP production in neurons (Yellen, 2018). Second, ATP production is tightly regulated by the brain’s energy demands through neurovascular coupling (Lund Madsen et al., 1999). A healthy state is therefore characterised by the brain’s ability to adapt to neuronal energy requirements. At rest, this entails oxygen supply driving the mitochondrial oscillator, with the mitochondrial and glycolytic oscillators operating in synchrony. During periods of increased energy demand, glycolysis can upregulate, with glucose supply assuming a prominent role. In all cases, synchronisation between glycolytic and mitochondrial oscillators is essential for maintaining neuronal health. A transient unsustainable uncoupling takes place as the system transitions between states. Each of these metabolic modes are demonstrated in Figure 2, with the solid arrows representing synchronisation between components.

Specifically, the instantaneous phase difference is defined as $\Delta \phi \left(t\right)=\phi_{\text{GO}}\left(t\right)-\phi_{\text{MO}}\left(t\right)$ . Phase synchronisation is defined here as 1:1 phase locking, characterised by a bounded and approximately constant Δϕ(t) with minimal phase drift over the latter half of the time series.

At low energy requirements, the neuron mainly produces ATP via oxidative phosphorylation (Figure 2A). As cognitive load increases, glycolysis is transiently upregulated to meet demand (Figure 2C) (Díaz-García and Yellen, 2019). Between these two regimes, a transitional region occurs (Figure 2B) in which each metabolic oscillator becomes phase locked to its corresponding supply oscillator (e.g., glycolysis to glucose availability and oxidative phosphorylation to oxygen availability), while the metabolic oscillators themselves are not synchronised.

**FIGURE 3:**
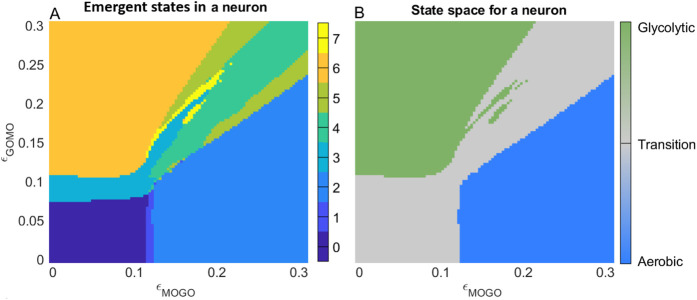
Phase synchronisation states for the model of healthy metabolic brain states at rest. **(A)** Each synchronisation state defined in Table 2 is assigned a colour. **(B)** Simplified version of the parameter space, assigning green (glycolytic), blue (oxidative), and grey (transition) colours to each metabolic mode.

By categorising the several possible synchronisation states into three distinct regimes we can drastically simplify the parameter space. Figure 3. illustrates this simplification across a range of coupling strengths between the metabolic oscillators.

The aerobic (blue), transitional (grey) and glycolytic (green) regions in Figure 3 are thus biologically relevant and easy to interpret. To delineate the synchronisation patterns into distinct groups, the phase synchronisation is evaluated. Table 2 indicates the different synchronisation states and assigns a colour, all of which correspond to Figure 3A. This framework simplifies the many represented regimes into a few biologically relevant regions.

**TABLE 2:** Synchronisation states corresponding to Figure 3A. An X in a box indicates synchronisation between the two oscillators. The final column indicates the metabolic mode for that synchronisation regime.

Number	Glu - GO	GO - MO	Oxy - MO	GOa - MO	Glu - GOa	State
0	X	​	X	​	X	Transition
1	​	​	X	​	X	Transition
2	​	X	X	​	X	Aerobic
3	X	​	​	​	X	Transition
4	​	​	​	​	X	Transition
5	​	X	​	​	X	Transition
6	X	X	​	X	X	Glycolytic
7	​	​	​	X	X	Glycolytic

For interpretation, the axes in Figure 3 show the range of coupling strengths between metabolic and supply oscillators. By selecting coupling strengths from Table 1 which correspond to a resting state, one may identify the synchronisation regime corresponding to resting-state dynamics.

#### 3.2.1 Coupling strengths during energy demand

Both neuronal glycolysis and oxidative phosphorylation require a continuous supply of glucose and oxygen (Doubovikov and Aksenov, 2020; Mergenthaler et al., 2013), so the corresponding supply couplings $\epsilon_{\mathrm{OxMO}}$ and $\epsilon_{\mathrm{GluGO}}$ are set relatively high, ensuring synchronization between metabolic oscillators and their supply. In contrast, astrocytes can draw on glycogen stores during periods of increased demand (Deitmer et al., 2019), necessitating a relatively weak coupling between the glucose supply and astrocyte $\left(\epsilon_{\mathrm{GluGOa}}=0.025\right)$ .

The role of astrocytic glycolysis in neuronal metabolism is debated (Díaz-García and Yellen, 2019; Bonvento and Bolaños, 2021), with some evidence suggesting only a supporting role under basal conditions. Accordingly, the astrocyte-to-neuron coupling $\epsilon_{\mathrm{GOaMO}}$ is kept low at rest. Following stimulation, however, lactate shuttling from astrocytes to neurons increases (Karagiannis et al., 2021; Kim et al., 2025), and the corresponding coupling rises (Mason, 2017; Zhang et al., 2025).

At low energy demand, the influence from the mitochondrial to the glycolytic oscillator is $\epsilon_{\mathrm{MOGO}}\approx 0.2$ , whereas the reverse coupling is $\epsilon_{\mathrm{GOMO}}\approx 0.1$ (Figure 3), consistent with an aerobic resting state in which most ATP is produced via mitochondrial pathways (Song et al., 2024).

#### 3.2.2 Dynamic changes with increased demand

**FIGURE 4:**
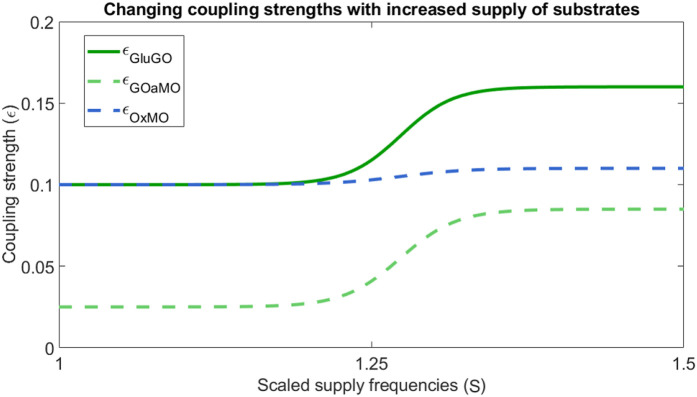
Dependence of coupling strengths $\varepsilon_{\mathrm{GluGO}}$ , $\varepsilon_{\mathrm{GOaMO}}$ , and $\varepsilon_{\mathrm{OxMO}}$ on substrate supply frequency. The x -axis shows the factor scaling the supply frequency ω , while the y -axis indicates the corresponding coupling strengths ε . Each curve represents how a specific coupling varies with increased substrate supply: $\varepsilon_{\mathrm{GluGO}}$ for Glu-GO, $\varepsilon_{\mathrm{GOaMO}}$ for GOa-MO, and $\varepsilon_{\mathrm{OxMO}}$ for Ox-MO. The supply frequency is scaled relative to the baseline values $\omega_{glu}=\left(\frac{2\pi }{200}\right),\omega_{ox}=\left(\frac{2\pi }{100}\right)$ .

The neurovascular unit is a highly nonautonomous system that adapts dynamically to meet fluctuating energy demands (Yellen, 2018; McConnell and Mishra, 2022). Understanding the interactions between its metabolic components is key to characterising the overall state of the system. Given the brain’s inherent metabolic flexibility, which is vital for supporting plasticity and cognition (Watts et al., 2018), it is insufficient to consider only the resting state. Here, activity-dependent stimulation is modelled by increasing the supply frequencies $\left(\omega_{\mathrm{Glu}}\;\omega_{Ox}\right)$ using a scale factor (S) between 1 and 1.5. The supply frequencies of oxygen and glucose scale linearly according to this factor S. For each parameter set, the resulting synchronisation states—and therefore the interactions between components—are analysed. As supply frequencies increase, mimicking enhanced vascular support during periods of activity, the coupling from vasculature to neuron strengthens, reflecting adaptation to higher energy requirements. Coupling strengths between oscillators were modulated using a sigmoidal function of the driving input, reflecting saturating responses of metabolic and neurovascular subsystems. This formulation ensures that weak inputs produce minimal coupling, while strong inputs approach maximal effective interaction, allowing smooth, nonlinear transitions in the system dynamics. Figure 4 shows how these coupling strengths evolve with increasing supply frequency.

The dependence of couplings on substrate supply frequency is illustrated in Figure 4. The coupling strength is shown as a function of the factor scaling the frequencies $\omega_{Glu}=\left(\frac{2\pi }{200}\right)$ and $\omega_{Ox}=\left(\frac{2\pi }{100}\right)$ . The Glu-GO coupling is represented as $\varepsilon_{\mathrm{GluGO}}$ , the GOa-MO coupling is represented as $\varepsilon_{\mathrm{GOaMO}}$ and the Ox-MO coupling as $\varepsilon_{\mathrm{OxMO}}$ . As the substrate supply frequency increases, the coupling strengths also rise to meet elevated energy requirements. Glucose supply upregulates more strongly than oxygen supply, since glycolysis—rather than OXPHOS—is preferentially increased during high energy demand. Additionally, the influences between the metabolic oscillators changes as demand increases. The influence flips, such that $\epsilon_{\mathrm{MOGO}}\approx 0.1\to 0.2$ , and $\epsilon_{\mathrm{GOMO}}\approx 0.2\to 0.1$ as glycolysis dominates the system to meet energy demand.

#### 3.2.3 Metabolism in dementia

Metabolic dysfunction is increasingly recognised as a key driver in the onset and progression of dementia (Craft, 2009). Neuronal processes rely on efficient energy metabolism, and impairments to this system can lead to widespread neuronal damage (Liu et al., 2025). Consequently, targeting metabolic abnormalities is emerging as a promising therapeutic strategy for neurodegenerative disease (Han et al., 2021).

In neurodegenerative disorders, vascular pathology—including altered haemodynamics, angiogenesis, endothelial degeneration, reduced vessel coverage, and compromised blood–brain barrier integrity—can impair substrate delivery (Govindpani et al., 2019; Torre, 2018). These changes disrupt the same coupling pathways that adapt to support healthy brain function, potentially initiating a feedback loop of worsening neuronal, metabolic, and vascular dysfunction. Ageing and dementia further impair cerebral blood flow regulation during cognitive activity (Sorond et al., 2008), largely through breakdowns in neurovascular coupling between neuronal activity and oxygen delivery (Bjerkan et al., 2025). Chronic cerebral hypoperfusion, often resulting from small vessel disease, stroke, or hypertension, is a common consequence (Duncombe et al., 2017).

In dementia, glucose delivery is compromised both by vascular dysfunction and by reduced expression of key transporters, including GLUT1 and GLUT3, as well as impaired astrocytic lactate transport through decreased monocarboxylate transporter (MCT) expression (Shah et al., 2012; Albaik et al., 2024). Notably, GLUT3 expression is markedly diminished in the cerebral cortex of Alzheimer’s disease (AD) patients, contributing to impaired neuronal glucose uptake (Duran-Aniotz and Hetz, 2016; Kumar et al., 2022; Kyrtata et al., 2021). In our model, these observations are captured as a reduced coupling between the vascular glucose supply and the glycolytic oscillator $\left(\epsilon_{\mathrm{GluGO}}\right)$ , alongside a weakened astrocyte-to-neuron coupling $\left(\epsilon_{\mathrm{GOaMO}}\right)$ .

Mitochondrial function declines with ageing and in neurodegenerative diseases, including Parkinson’s disease, dementia with Lewy bodies, and AD (Navarro and Boveris, 2010). ATP production via oxidative phosphorylation is reduced (Boveris and Navarro, 2008), and cerebral oxygen utilisation can fall by up to 50% in patients with central nervous system disorders (Frackowiak et al., 1988). In AD, reduced cerebral blood flow limits oxygen delivery, impairing mitochondrial ATP synthesis and neuronal activity (Golpich et al., 2017; Liu et al., 2023). In the model, this is implemented as a reduction in the coupling between oxygen supply and the mitochondrial oscillator $\left(\epsilon_{\mathrm{OxMO}}\right)$ .

Neurovascular coupling normally enables dynamic adjustments of substrate delivery to match energy demand, a process known as functional hyperaemia (Iadecola, 2017; Cox et al., 1993). In dementia, this mechanism is impaired (Bjerkan et al., 2025; Kisler et al., 2017; Cai et al., 2017), contributing to metabolic inflexibility (Zhang et al., 2021). Imaging studies consistently demonstrate reductions in both oxygen and glucose delivery in affected individuals (Kumar et al., 2022; Tao et al., 2024).

In summary, dementia-related metabolic changes are represented in the model as reduced oxygen, glucose and lactate supply couplings. These changes reflect the cerebral hypoxia, impaired transporter expression and vascular delivery. therefore, we focus on substrate supply deficiencies to highlight their central role in dementia pathophysiology.

## 4 Results

The established model can now be manipulated to simulate metabolically active and pathological conditions. Dementia is strongly associated with disruptions in neuronal energy metabolism. In this context, two key dementia-related alterations are examined: reduced oxygen availability and impaired glucose/lactate supply. These changes allow investigation into how limited metabolic substrate availability affects the broader system dynamics. To represent these pathological scenarios, the coupling parameters governing substrate supply are reduced. However, before analysing these altered states, we first consolidate the behaviour of the healthy system by examining the parameter space during activation. Here, the previously defined parameters are applied to simulate both resting conditions and periods of stimulation.

### 4.1 Resting and active state changes in the brain

**FIGURE 5:**
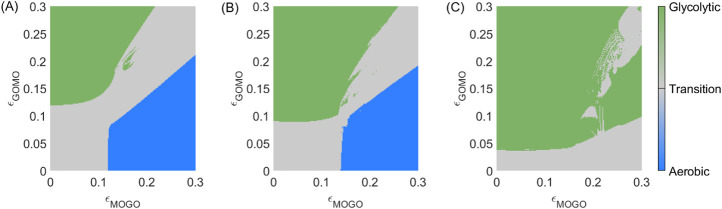
Transition between metabolic states in the healthy brain. **(A)** Resting state (S=1) . **(B)** Early activation phase (S=1.25) . **(C)** Fully activated state (S=1.5) .

In the resting state, metabolic components operate aerobically, with all oscillators synchronised to the oxygen supply. In this state, glycolysis acts in a supporting role, sufficient to provide substrates for oxidative phosphorylation. Increasing cognitive demand drives greater substrate delivery to the brain, modelled as an increase in ω , thereby inducing metabolic adaptations. Glucose supply is upregulated to supplement ATP production, ensuring that elevated energy demands are met. The capacity to shift flexibly between metabolic modes is a hallmark of a healthy brain. Figure 5 illustrates this progression, showing oscillator synchronisation shifting towards glycolytic modes as demand intensifies. Figure 5A represents the resting state with substrate supplies $\omega_{Ox}$ , $\omega_{\mathrm{Glu}}$ as in Table 1. Figure 5B shows the transitional state when substrate supply increases by a scale factor S=1.25 . Figure 5C depicts the glycolytic state required to meet high energy demand at S=1.5 , following the sigmoidal coupling transitions described in Figure 4. The values of S were selected to provide clear visual distinction in simulations while remaining physiologically plausible.

The phase space of the oscillators reveals the dominant metabolic influence at different vascular flow rates by mapping the synchronisation state across a range of coupling strengths between neuronal metabolic oscillators. As described previously, at low energy demand (Figure 5A), the influence from the mitochondrial to the glycolytic oscillator is $\epsilon_{\mathrm{MOGO}}\approx 0.2$ , while the reverse coupling is $\epsilon_{\mathrm{GOMO}}\approx 0.1$ . As energy demand increases (Figure 5C), this relationship reverses: $\epsilon_{\mathrm{MOGO}}\approx 0.1$ and $\epsilon_{\mathrm{GOMO}}\approx 0.2$ . Therefore the dominant metabolic mode switches from aerobic respiration to glycolysis as energy demand, and thus substrate supply, increases.

**FIGURE 6:**
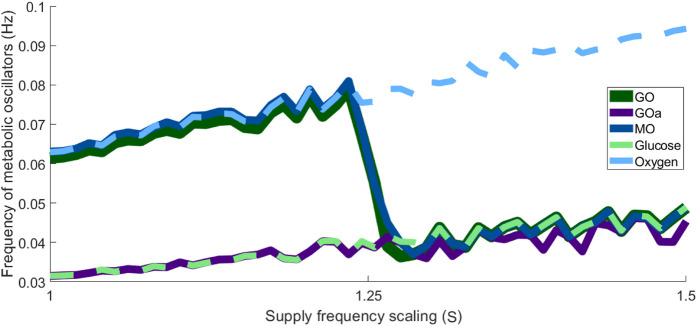
Dependence of the metabolic oscillators’ frequency on the scaled substrate frequency in the healthy state.

To clarify the dynamics further, one can express the frequencies of each oscillator across a continuos supply frequency scaling, derived from the phase evolution over time. As shown in Figure 6, the mitochondrial and glycolytic oscillators shift from synchronisation with the oxygen supply to synchronisation with the glucose supply as substrate frequency increases. This behaviour mirrors that of the healthy brain, where increased cognitive demand enhances haemodynamic flow, driving a metabolic shift towards glycolytic activity to meet elevated energy demand.

### 4.2 Impaired oxygen supply coupling

**FIGURE 7:**
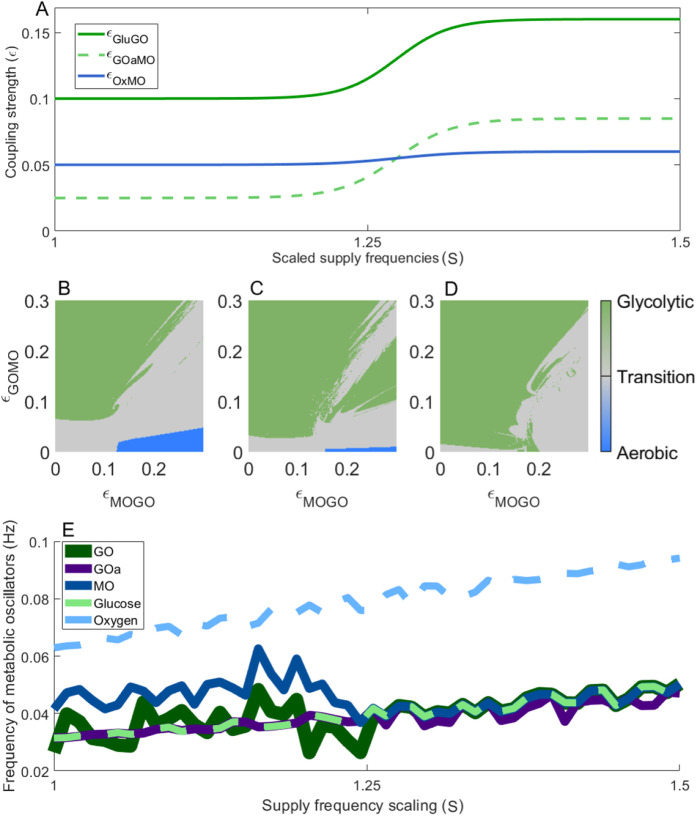
Phase space representation of the resting and active states when the coupling between oxygen and the mitochondrial oscillator is reduced, mimicking impaired oxygen delivery. **(A)** Changes in coupling strength under reduced oxygen supply to neuronal mitochondria. **(B–D)** Phase space trajectories at three scaled values of the supply frequency: 1 **(B)**, 1.25 **(C)**, and 1.5 **(D)**, representing resting, early activation, and fully activated metabolic states, respectively. **(E)** Frequency of the metabolic oscillators as energy demand increases, illustrating the transition from baseline to elevated metabolic activity.

As described in Section 3.2.3, oxygen delivery is disrupted by several factors in neurodegenerative diseases. To explore the consequences for metabolic oscillator interactions, the coupling strength from the vasculature to the mitochondria was reduced in both resting and activated states. This decrease is shown in Figure 7A, while the sigmoidal coupling transitions for lactate and glucose supply remained unchanged.

The phase spaces in Figures 7B–D show that dynamics are dominated by the glycolytic oscillator across all supply frequency scalings S = (1, 1.25 and 1.5), with no substantial aerobic region even in the resting state. This occurs because the oxygen and mitochondrial oscillators are effectively uncoupled: the reduced coupling strength cannot overcome the frequency mismatch, allowing glycolytic components to dominate. Figure 7E reinforces this, showing uncoupled behaviour at low energy demand and a shift to glucose supply as the primary driver at high demand. Although a transition to glycolytic metabolism still occurs under high energy demand, the absence of an aerobic mode at rest results in insufficient total ATP production.

### 4.3 Impaired glucose supply coupling

**FIGURE 8:**
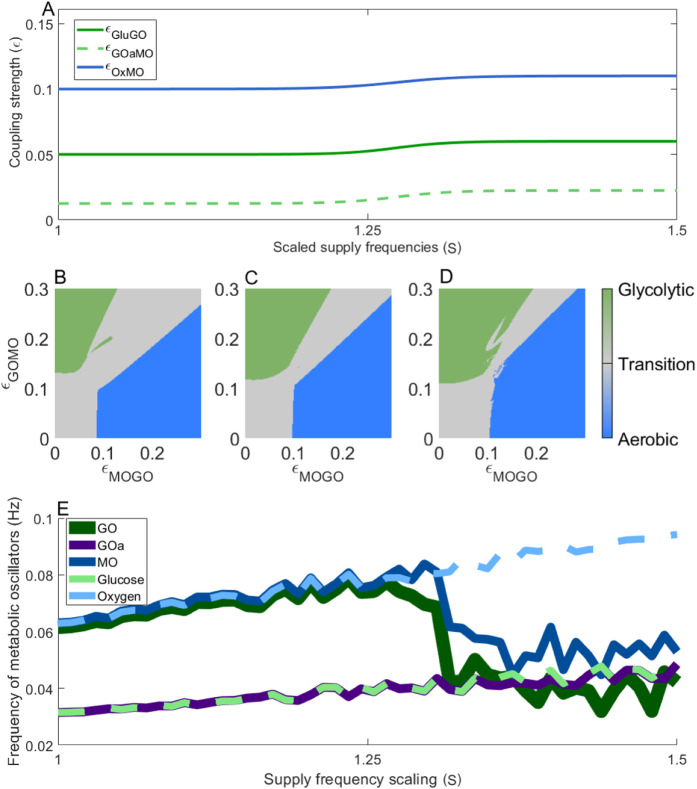
Phase space representation of the resting and active states when glucose and lactate supplies are compromised, to mimic reduced substrate availability as observed in dementia. **(A)** Changes in oscillator coupling strength under impaired glucose and lactate delivery to neuronal mitochondria. **(B–D)** Phase space trajectories at three scaled values of the supply frequency: 1 **(B)**, 1.25 **(C)**, and 1.5 **(D)**, corresponding to resting, early activation, and fully activated metabolic states, respectively. **(E)** Frequency of the metabolic oscillators as energy demand increases, illustrating the transition from baseline to elevated metabolic activity.

Glucose and lactate delivery to neurons are also reduced in neurodegenerative disease. To model this, both resting-state lactate and glucose supplies were halved. In addition, the large post-stimulation increase in glucose supply was substantially dampened, reflecting the impaired ability to dynamically adjust substrate intake in dementia. The resulting coupling strengths are illustrated in Figure 8A.

The phase spaces in Figures 8B–D highlight the reduced ability of metabolism to transition to a glycolytic state. Particularly, Figure 8D demonstrates how the glycolytic region fails to dominate the interaction during increased demand, unlike in the healthy case (Figure 5C). Impaired glucose supply therefore restricts the brain’s ability to adapt to increased neural load, likely preventing it from meeting metabolic demand during activity-dependent stimulation. This is reinforced by Figure 8E: although the aerobic state at rest is sufficient, as the supply frequency increase neither the mitochondrial or glycolytic oscillators synchronise with each other or with the glucose supply.

## 5 Discussion

Despite being based on only a few nonautonomous phase oscillators, the present phenomenological model captures the complex interactions underlying ATP production in the brain while requiring far fewer equations and parameters than mass-based approaches. It focuses on the neurovascular unit (NVU), whose role in pathogenesis is increasingly recognised (Bjerkan et al., 2025). Whereas previous models (Aubert and Costalat, 2005; Simpson et al., 2007) described concentrations of lactate, glucose, and other metabolites, they largely overlooked the intrinsically nonlinear and nonautonomous nature of these processes.

Here, multiple biochemical pathways and compounds are represented as coupled phase oscillators, enabling a qualitative characterisation of energy metabolism in which healthy and pathological states emerge as distinct synchronisation patterns. Rather than forcing oscillations to arise, oscillatory behaviour is built into the model from the outset, allowing direct analysis of qualitative dynamics and emergent phenomena. This framework offers several advantages: reduced complexity, fewer free parameters, an intrinsic resilience against noise (Rowland Adams et al., 2023), and a clear distinction between healthy and pathological states. While traditional models output continuous substrate concentrations (Mintun et al., 2001), our approach captures dynamic interplay through synchronisation phenomena, revealing essential features of brain energy metabolism despite its simplicity.

During task-driven or cognitively demanding states, the brain requires increased energy supply, resulting in a metabolic shift towards glycolytic activity and increased haemodynamic flow. Cerebral blood flow and glucose uptake increase during neuronal activation, enabling rapid ATP production (Sorond et al., 2008; Neil Vaishnavi et al., 2010).

The model reproduces key pathological features. Reduced glucose and lactate availability induced metabolic inflexibility, limiting transitions to glycolysis under increased demand. Similarly, reduced oxygen supply impaired oxidative phosphorylation, forcing reliance on glycolysis. These behaviours mimic NVU disruptions in dementia, which constrain ATP production and impair adaptation to neural load. This mechanism ensures that neuronal activity is matched by local metabolic supply in healthy brains. Metabolic abnormalities in dementia—including shifts toward glycolysis linked to impaired insulin signalling, cardiovascular dysfunction and mitochondrial dysfunction (Yan et al., 2020; Meng et al., 2025)—have led some to describe Alzheimer’s disease as “type three diabetes” (Meng et al., 2025). Additionally, suboptimal ATP availability is increasingly recognised as a key driver of neural dysfunction (Ebanks et al., 2020; Aran and Singh, 2023), reflecting the tight coupling between energy supply and neural activity (Simon, 2001).

While in this work we focus upon dementia, the framework can be used to explain the effect of ageing, or can be extended to other neurological conditions such as epilepsy or autism spectrum disorder. Additionally, the framework can be made specifically for cases like Alzheimer’s, Huntington’s or Parkinson’s disease.

Future iterations of the model can refine parameters as experimental evidence accumulates. The small parameter set facilitates such updates, while the current framework provides a foundation for modelling neuronal energetics. One simplification here was treating energy demand as a unidirectional vascular influence, yet neurovascular coupling is bidirectional, with neuronal activity also shaping blood flow (Zhong et al., 2025; Kaplan et al., 2020). We also note that the model does not include explicit ATP dynamics, pH or ion homeostasis, and simplifies multi-step metabolic pathways and the neurovascular unit to five oscillators. These choices were made to focus specifically on the metabolic interactions and substrate-driven oscillatory transitions. Extending the model to incorporate this reciprocity, and introducing oscillator networks as in (Rowland Adams and Stefanovska, 2021), will enable the spatial complexity of neuronal energetics to be captured. Similarly, models of higher order connectomes, or digital twins of human brain function (e.g., (Jirsa et al., 2023; Santoro et al., 2024)) can benefit from including the framework proposed here.

The strength of this model lies in its simplicity: it reproduces essential markers of diseased states while remaining tractable. Further development, guided by *in vivo* data of the relevant natural frequencies, will enhance its ability to recreate the complex metabolic behaviours of the brain. By focusing explicitly on metabolism, this work provides a first step towards deeper understanding of neurodegenerative disorders.

## 6 Summary

A model of interacting nonautonomous phase oscillators is introduced to qualitatively capture the metabolic state within the neurovascular unit and its alterations in dementia. Parameters represent dynamic interactions between oscillatory metabolic processes, with the nonautonomous phase dynamics framework (Kloeden and Rasmussen, 2011; Suprunenko et al., 2013; Lancaster et al., 2016; Rowland Adams et al., 2023) offering a tractable alternative to mass-based models of metabolite transport and chemical reactions.

The model distinguishes between healthy and pathological brain states: parameters defining the healthy state yielded synchronised metabolic oscillations that supported optimal ATP production, whereas dementia-related changes reproduced realistic pathological scenarios. By emphasising the role of synchrony within the neurovascular unit, the phase-oscillator approach captures essential features of real biological systems.

This model should be regarded as a first step towards a more comprehensive framework involving networks of oscillators. Further experimental validation of parameter choices will be required, but even in its current form the model offers a simple yet powerful means of representing brain metabolic processes in health and disease. Framing the brain as fundamentally dependent on nutrient supply may ultimately provide a clearer understanding of the metabolic shifts that drive dementia.

## Data Availability

Codes used in this study are available on Github at: https://github.com/SamJKBarnes/Modelling-brain-metabolism-with-interacting-nonautonomous-phase-oscillators.
